# Use of target vessel ballooning to facilitate endovascular treatment in the case of branched endovascular aneurysm repair with a retrograde approach

**DOI:** 10.1016/j.jvscit.2023.101330

**Published:** 2023-09-22

**Authors:** Fabio Riccardo Pisa, Giovanni Spinella, Bianca Pane, Giovanni Pratesi

**Affiliations:** a Department of Surgical and Integrated Diagnostic Sciences, University of Genoa, Genoa, Italy; b Vascular and Endovascular Surgery Unit, IRCCS Ospedale Policlinico San Martino, Genoa, Italy

**Keywords:** Branched EVAR, Endovascular procedures, Retrograde approach, Thoracoabdominal aortic aneurysm

## Abstract

A case of a new technique for branched endovascular aneurysm repair with a retrograde approach and ostial stenosis of the target vessel is reported. An angioplasty balloon was placed within the target vessel and used to give added stability to catheter advancement to place the stiff guidewire needed for placement of a bridging stent graft. In brief, a standard guidewire was first placed inside the target vessel through the retrograde approach. Next, the balloon was placed from outside the stent graft, again through a contralateral retrograde approach. Then, the angioplasty balloon was inflated, and a support catheter was advanced to the balloon and then slowly deflated to allow the catheter to advance. Finally, the stiff guidewire was placed. Subsequently, the bridging stent was placed and deployed. This technique is feasible and can be used in selected cases to use a retrograde approach when ostial stenosis of the target vessel is present.

Endovascular treatment of thoracoabdominal aneurysms using a fenestrated or branched endoprosthesis is a valid option that is increasingly being used.^1^ The target vessels used for branched endovascular aneurysm repair (B-EVAR) cannulation and stenting and their patency postoperatively and during follow-up continue to be a critical issue.^2^, ^3^, ^4^, ^5^ The best bridging stent approach during B-EVAR procedures continues to be debated. Many investigators prefer upper extremity access to catheterize antegrade branches and their target vessels.^6^^,^^7^ However, numerous complications, including stroke, the formation of pseudoaneurysms or intramural hematomas, and distal embolization, are associated with this type of access.^8^, ^9^, ^10^ To prevent these complications, investigators have proposed different techniques using a retrograde approach through the femoral artery.^11^^,^^12^ Despite standardization of a retrograde approach, the anatomy of the renal arteries plays a fundamental role in the technical success of the procedure.^13^^,^^14^ Another challenge in retrograde cannulation can be the presence of ostial stenosis of the target vessels.^14^^,^^15^ The retrograde approach is a novel treatment that, with the addition of procedures such as ballooning target vessels, might allow for successful procedural completion.

Therefore, we report the case of a patient with a type II thoracoabdominal aneurysm, according to the Crawford classification, with left renal stenosis characterized by an upward course. The patient underwent B-EVAR in which a new technique of selective cannulation of the target vessel was used.

## Case report

A 69-year-old male patient presented with an incidentally found asymptomatic thoracoabdominal aneurysm. The patient's clinical history revealed hypertension, smoking, and obesity, with no surgical history. The patient was prescribed acetylsalicylic acid for primary cardiovascular prevention. Preoperative computed tomography angiography showed a thoracoabdominal type II aneurysm. No adequate proximal landing zones distal to the subclavian artery were observed. In addition, the presence of ostial stenosis of the left renal artery was revealed, 2 mm from the origin of the vessel, which had induced a 65% stenosis for 5.5 mm exhibiting an upward course in the proximal segment. The aortic diameter in the plane passing through the takeoff of the renal arteries was 46 mm. Given the complexity of the lesion, the patient underwent multistep thoracoabdominal EVAR. The endovascular procedures were planned by the same surgical team that performed the intervention.

**Fig 1:**
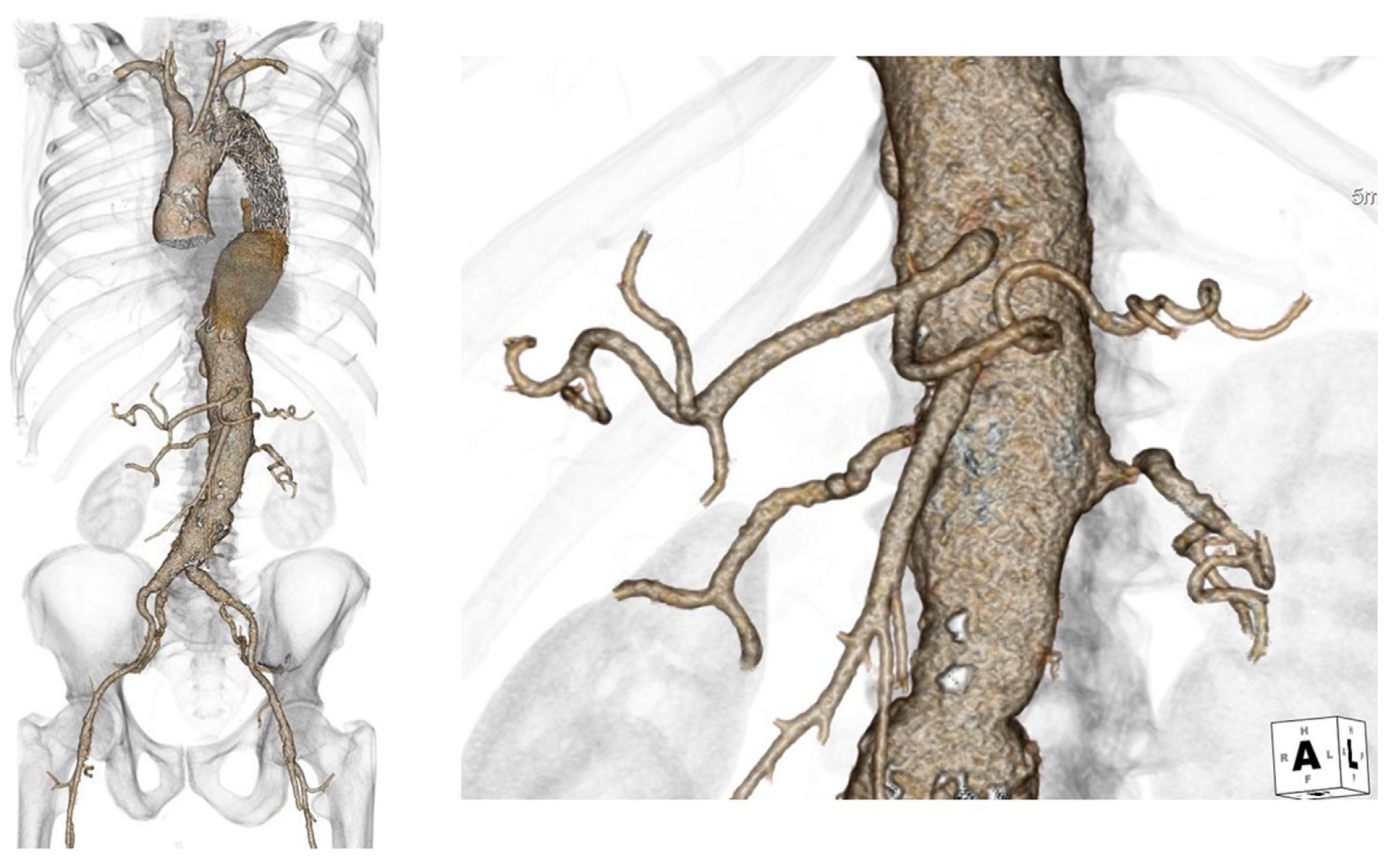
Preoperative computed tomography angiogram showing ostial stenosis and hostile orientation of the left renal artery.

The first step of the endovascular procedure was implantation of two thoracic modules with a proximal landing in zone 2, according to Ishimaru, continuing distally to the left common carotid artery origin with a distal landing zone just above the celiac trunk, and in situ fenestration for the left subclavian artery. The procedure was completed with deployment of a covered stent in the fenestration site at the level of the left subclavian artery (Fig 1).

**Fig 2:**
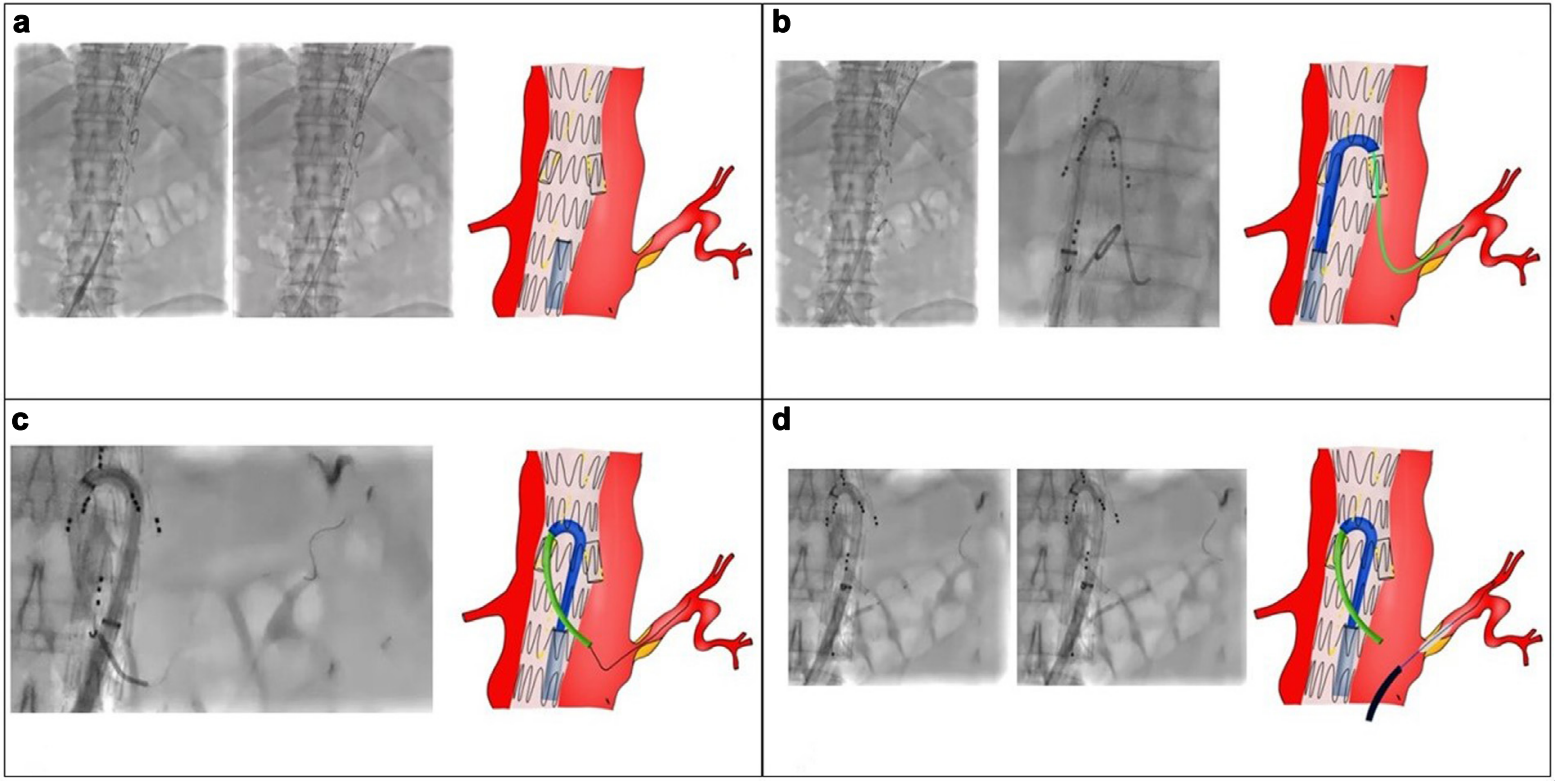
**A,** DrySeal 18F introducer (W.L. Gore & Associates) insertion after branched module release and delivery system recovery. **B,** Positioning of Heli-Fx 22-mm catheter (Medtronic Vascular) and Flexor 7F, 45-cm guiding sheath (Cook Medical Inc) at the prosthetic branch for the left renal artery. Selective cannulation of the left renal artery using a Bern 5F, 65-cm catheter (Boston Scientific) and standard Terumo guidewire (Terumo Medical). **C,** Repositioning of the steerable catheter at the prosthetic branch of the right renal artery, and selective cannulation attempt with the diagnostic catheter and Terumo guidewire. Once the guidewire was advanced, the catheter was pushed out of the vessel, along with the guidewire itself. **D,** Flexor 7F, 45-cm guiding sheath (Cook Medical Inc) positioning and advancement up to the ostium of the left renal artery. After exchange of the 5F introducer for a 8F introducer from the left femoral access, cannulation with the V18 guidewire and predilatation with an ultrasoft 5 × 20-mm balloon was performed.

**Fig 3:**
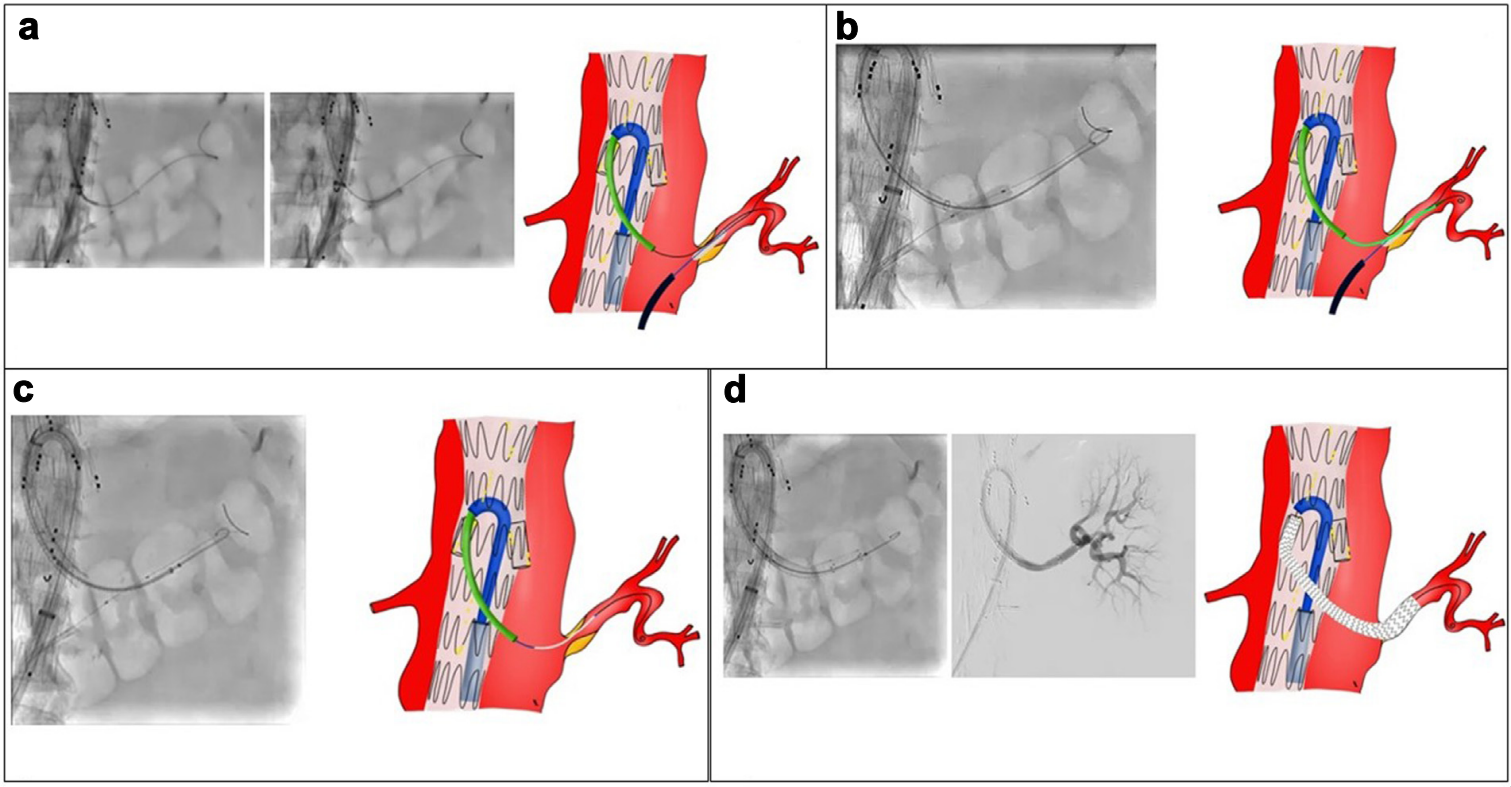
**A,** Bern catheter (Boston Scientific) position corresponding to the ostium of the left renal artery with cannulation of the target vessel with a Terumo guidewire by modulating inflation of the previously positioned balloon and fixation of the guidewire inside the left renal artery by inflating the ultrasoft balloon. **B,** Bern catheter exchange with a CXI 0.035-in. catheter (Cook Medical), crossing of the stenosis by slightly deflating the balloon, and successful guidewire exchange (260-mm Rosen for a Terumo guidewire). **C,** Stenting of the target vessel with Viabahn self-expandable stents (W.L. Gore & Associates) on the Rosen guidewire (Cook Medical) facilitated by modulation of the inflation of the balloon. **D,** Final result of stenting of the hostile left renal artery.

**Fig 4:**
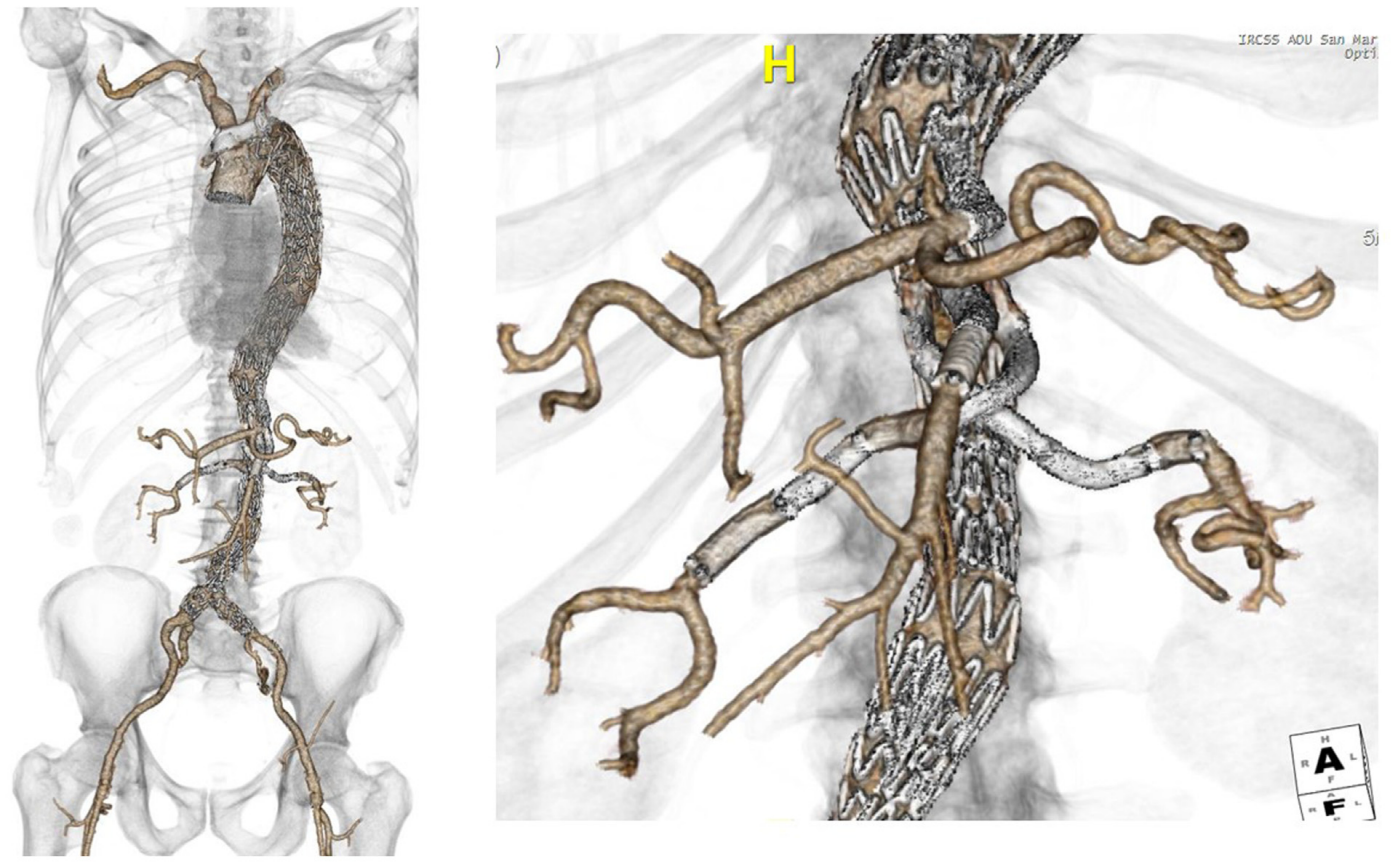
Postoperative computed tomography angiogram showing completion of the three-step treatment.

The second endovascular step was scheduled ∼4 months after the first step and involved placement of a branched endoprosthesis (t-Branch; Cook Medical Inc) and bridging stenting of the visceral vessels with placement of a bifurcated abdominal graft scheduled. Bilateral percutaneous femoral access was obtained. On the right side, two ProGlide vascular closure devices (Abbott Vascular) were preimplanted with the insertion of an 11F introducer on a standard Terumo guidewire (Terumo Medical). From the left, after positioning a 5F introducer, a pigtail 5F diagnostic catheter was advanced, and preoperative angiography was performed to identify the visceral vessel origin and align the fusion computed tomography scan. From the right, after exchanging the guidewire with a Lunderquist 260 mm (Cook Medical Inc) and confirming correct alignment with the visceral vessel origin, the branched endoprosthetic module was inserted, with sequential release of the visceral branches. A complete branched module release and delivery system recovery were performed. On the right side, an 18F DrySeal introducer (W.L. Gore & Associates) was exchanged. After exchanging the Lunderquist guidewire with a Terumo standard guidewire (Terumo Medical), the Heli-Fx 22-mm catheter (Medtronic Vascular) and a Flexor 7F, 45-cm guiding sheath (Cook Medical Inc) were advanced and positioned at the prosthetic branch for the left renal artery. A Bern 5F, 65-cm catheter (Boston Scientific) was inserted, and an attempt at selective cannulation of the left renal artery was performed with a standard Terumo guidewire (Terumo Medical). After successful placement of the guidewire in the artery, attempts to stabilize the system by advancing the catheter were unsuccessful owing to resistance from the ostial stenosis and angulation of the renal artery. Furthermore, because of the pressure exerted on the ostium of the vessel, correct positioning of the guidewire was lost. A second engagement was attempted and was unsuccessful. A third attempt was made by exchanging the Bern catheter for the 5F, 65-cm vertebral hydrophilic catheter. Selective cannulation of the left renal artery was performed. However, during the exchange of the Terumo standard guide (Cook Medical Inc) with a Rosen 260-mm guidewire (Cook Medical Inc), owing to the lack of sufficient stability, the catheter was pushed to the proximal edge of the vessel. The Rosen guidewire was switched for a standard Terumo guidewire to reposition the catheter. Once the guidewire was advanced, the catheter was pushed out of the vessel along with the guidewire itself. The steerable catheter was repositioned at the prosthetic branch of the right renal artery. Two attempts at selective cannulation of the left renal artery with a standard Terumo guidewire and 5F Kumpe (Cook Medical Inc) and vertebral hydrophilic catheter, respectively, were performed without success. At this point, from the left femoral artery access, after removing the 5F pigtail catheter, the 5F introducer was exchanged for an 8F introducer and the Flexor 7F, 45-cm guiding sheath (Cook Medical Inc) was advanced up to the ostium of the left renal artery. After advancement of the V18 guidewire (Boston Scientific), selective cannulation of the vessel was performed. An ultrasoft, 5 × 20-mm balloon was then placed in the left renal artery, and, after crossing the ostial lesion, angioplasty was performed (Fig 2). The previously positioned inflated balloon was left in place. On the right, the Bern catheter (Boston Scientific) was advanced inside the guiding sheath and was positioned a few millimeters from the origin of the left renal artery up to the ostium of the vessel. The balloon positioned in the left renal artery was slightly deflated and, with a standard Terumo guidewire (Terumo Medical), the vessel was cannulated. After passage of the guidewire, the balloon was inflated again, and the guidewire was fixed. The Bern catheter was exchanged with a CXI 0.035-in. catheter (Cook Medical Inc). By modulating the inflation of the balloon, the stenosis was crossed, and the catheter was advanced into the distal renal artery. With the balloon inflated, the Terumo guidewire (Terumo Medical) was exchanged for the Rosen 260-mm guidewire (Cook Medical Inc), and the balloon-expandable BeGraft 6 × 58-mm stent (Bentley InnoMed GmbH) was advanced with proximal release inside the branch of the right renal artery of the branched endoprosthesis. The bridging stent of the left renal artery was completed with placement of two more Viabahn self-expandable stents (6 × 100 mm and 6 × 50 mm; W.L. Gore & Associates), with the balloon deflated to allow for correct placement of the stents (Fig 3). The procedure was completed with bridging stent placement of the remaining visceral vessels, implantation of the bifurcated Unibody 22-81 stent (Cook Medical) adequately imbricated, branching of the endoprosthetic component, and placement of the Zenith Alpha spiral Z 20-42 right iliac leg (Cook Medical Inc). The completion angiogram at the end of the procedure documented correct positioning of the branched endoprosthesis and bridging stents, normal patency of the aortic endoprosthesis, no signs of kinking and/or dissection in the stenting site of the visceral vessels, and normal patency of the femoral iliac axes with reperfusion of the aneurysmatic sac from the gate of the forked body. The fluoroscopy time was 178 minutes for the procedure. The dose ∙ area product was 800 Gy/cm^2^. The postoperative course was normal without any complications. The third step of endovascular aneurysm repair was performed 2 months after the second step using a Zenith Alpha 16-77 left iliac limb (Cook Medical Inc; Fig 4).

## Discussion

The best access strategy for bridge stenting in B-EVAR procedures is currently a critical issue. Since the early origins of aortic endovascular treatment, the anterograde approach through the axillary or brachial artery has always been recommended. However, choosing to adopt this technique when an important parietal thrombus is present exposes the patient to the risk of significant complications, such as embolization, during aortic arch navigation. Embolization can present as stroke in the event of the mobilization of material toward the epiaortic vessels, intramural hematoma formation, or aortic dissection.^15^^,^^16^ Furthermore, complex anatomy such as a type III aortic arch and/or the presence of calcifications in the epiaortic vessels or aorta itself, coupled with previous interventions such as debranching or a frozen elephant trunk procedure, could represent factors contributing to technical failure or be contraindications for this type of approach.^8^^,^^17^^,^^18^ For these reasons and with the development of steerable catheters during the past few years, retrograde cannulation with a femoral approach has been proposed.^8^^,^^9^^,^^19^

In the reported case, the retrograde approach was chosen for two essential reasons. The first reason was linked to the multistage procedure with the first step of thoracic EVAR and in situ fenestration for the left subclavian artery. In the present case, antegrade access from the right was not recommended and was contraindicated on the left owing to the risk of compromising the previous implant. The second reason was linked to standardization of the bridging stent technique we have developed at our center, which provided retrograde access using a steerable catheter for cannulation of the branches dedicated to the visceral vessels, where possible. Moreover, the anatomy of the target vessels plays a fundamental role in choosing which approach to use.

In the reported case, the takeoff and course of the left renal artery with ostial stenosis probably made the bridging stent technique more complex with the use of an antegrade approach from above owing to its course and upward inclination in the proximal segment. In addition, given the aortic diameter in the plane passing through the origin of the renal arteries (46 mm), we decided not to use a custom-made fenestrated endoprosthesis because the distance between the fenestration and target could have been too long to cover without increasing the risk of bridging stent instability. Therefore, we decided to use a branched off-the-shelf endograft. The main issue related to bridging stent placement for the left renal artery with the reported anatomic characteristics was related to the lack of stability in the vessel engagement phase. Angulation of the renal artery and the ostial stenosis hindered advancement of the catheter and led to the loss of positioning of the guidewire inside the vessel. Passage of the catheter induced straightening of the guidewire with consequent induction of traction toward the outside of the vessel, which resulted in the exit of the guidewire itself. To manage this, we decided to engage the left renal artery by positioning the steerable catheter in the contralateral branch for the right renal artery to reduce the angle of incidence between the catheter and the ostium of the vessel. However, this did not resolve the issue. With the impossibility of achieving a stable position with the guidewire within the target vessel for catheter advancement through the renal artery branches, we engaged the vessel from outside the endograft to perform predilatation with a low-profile balloon. Positioning of the balloon and modulation of the inflation made it possible to facilitate passage of the catheter and stiff guidewire and anchor it inside the artery. The stability of the system obtained with this technique facilitated passage of the catheter inside the vessel by controlling and contrasting the traction induced by the catheter itself on the guidewire with the balloon, guaranteeing the maintenance of advancement.

## Conclusions

This technique for retrograde cannulation during B-EVAR, with maintenance of adequate stability and advancement by modulation of the balloon within the target vessel, has been shown to be feasible in some complex visceral vessel anatomies.

## Disclosures

None.
